# Analysis of Related Metabolites Affecting Taste Values in Rice under Different Nitrogen Fertilizer Amounts and Planting Densities

**DOI:** 10.3390/foods11101508

**Published:** 2022-05-22

**Authors:** Qiangqiang Xiong, Changhui Sun, Hong Shi, Shuo Cai, Hengwang Xie, Fangping Liu, Jinyan Zhu

**Affiliations:** 1 Jiangxi Irrigation Experiment Central Station, Nanchang 330201, China; qqxiong@yzu.edu.cn (Q.X.); 8659979@163.com (H.S.); caishuo0911@163.com (S.C.); xhw2208@163.com (H.X.); fpl202204@163.com (F.L.); 2 Jiangsu Key Laboratory of Crop Genetics and Physiology/Jiangsu Key Laboratory of Crop Cultivation and Physiology, Agricultural College, Yangzhou University, Yangzhou 225009, China; schanghui2022@163.com; 3 Jiangsu Co-Innovation Center for Modern Production Technology of Grain Crops, Agricultural College, Yangzhou University, Yangzhou 225009, China

**Keywords:** nitrogen fertilizer, planting density, rice quality, metabolome, taste value

## Abstract

The aim of this study was to explore the differences in metabolites related to rice quality formation under different nitrogen (N) fertilizers and planting densities. In this study, Yangnongxiang 28 was used as the experimental material with the following conditions: high nitrogen and low density (HNLD; high nitrogen: 360 kg·hm^−2^, low density: the row spacing of rice plants was 16 cm × 30 cm), medium nitrogen and medium density (MNMD; medium nitrogen: 270 kg·hm^−2^, medium density: the row spacing of rice plants was 13 cm × 30 cm), and low nitrogen and high density (LNHD; low nitrogen: 270 kg·hm^−2^, high density: the row spacing of rice plants was 10 cm × 30 cm). The rice quality indexes, including the processing quality, amylose content, and taste value, were compared under different treatments, and we analyzed their relationship with the metabolites. The results show that the milled rice rate of HNLD was 13.85% and was 1.89% higher than that of LNHD and MNMD, respectively. The head milled rice rate of HNLD was 32.45% and 6.39% higher than that of LNHD and MNMD, respectively. The milled rice rate and head milled rice rate of HNLD and MNMD were significantly higher than those of LNHD. This study identified 22 differential metabolites (DMs) in HNLD and LNHD, 38 DMs in HNLD and MNMD, and 23 DMs in LNHD and MNMD. Most of the identified differential metabolites were lipid metabolites, which were mainly enriched in the lipid metabolic pathways and amino acid metabolic pathways. The correlation analysis showed that the lipid metabolite physapubescin was significantly negatively correlated with the taste value. The lipid metabolites 2-undecen-1-ol, lucidenic acid F, and 8-deoxy-11,13-dihydroxygrosheimin were significantly positively correlated with the taste value. Lipids may be important substances that lead to differences in taste under different nitrogen fertilizer and density treatments.

## 1. Introduction

With the development of China’s economy and the improvement in people’s living standards, higher requirements have been placed on the eating quality and nutritional quality of rice. On the premise of ensuring grain output, the high-quality rice industry has become an inevitable choice for long-term development [1]. Therefore, it is important to study the composition and formation mechanism of rice quality. Rice quality is affected not only by genetic characteristics, but also by environmental conditions and cultivation methods [2,3]. Nitrogen fertilizer management and transplanting density are the main cultivation techniques used during the process of rice production. There are many domestic and foreign studies on the effects of nitrogen fertilizer and transplanting density on rice growth and rice quality [4,5]. However, there are few studies on the formation of rice quality under nitrogen fertilizer and planting density combined with metabolomics.

Metabolomics is a discipline that identifies and quantitatively analyzes the composition of metabolites in specific tissues or cells of an organism, which can reveal the biological function of an organism from a metabolic perspective and interpret life phenomena [6,7]. With the continuous development and improvement of omics technology, metabolomics technology plays an increasingly important role in the detection and analysis of the nutritional quality of crop products [8,9]. The nutrient quality and metabolism databases of major crops, such as rice, have been established one after another, and the types, content differences, and metabolic synthesis pathways of various nutrients in crops have been systematically studied [10,11,12]. Not only can metabolites in rice grains fully reflect the overall metabolic state of rice seeds [13], but also the types and contents of metabolites have an important impact on rice quality. Studies have used metabolomic techniques to explore the nutritional diversity of three major crops (wheat, rice, and maize) and three fruits (grape, banana, and mango). The metabolic diversity of three major food crops and three fruits was clarified, complementary patterns of nutrient accumulation among different species were revealed, and species-specific patterns of bioactive compounds were deciphered [14]. Metabolomics can detect the differences in small molecule metabolites in agricultural products at the biological level and can qualitatively and quantitatively analyze the differences in the nutritional components of crops under different varieties and different growth conditions [15]. We previously proved that the contents of flavonoids and phenol metabolites are strongly correlated with the total antioxidant capacity and screened out six candidate metabolite markers that can be used as the antioxidant capacity of glutinous rice grains [16]. In order to explore the differences in the metabolites of Yangnongxiang 28 under different nitrogen fertilizers and planting densities and their relationship with the formation of rice quality, we carried out three treatments: high nitrogen and low density (HNLD), medium nitrogen and medium density (MNMD), and low nitrogen and high density (LNHD). Untargeted metabolomics is an unbiased and simultaneous detection and analysis technique for all small molecule metabolites. By comparing the metabolite differences between different samples and screening the key differential metabolic pathways, the metabolism-related processes of organisms are further elucidated. Therefore, metabolomics is more and more widely used in the fields of plant growth and development, crop quality formation, and so on. In the study, untargeted metabolomics technology was used to identify metabolites in milled rice and to select possible markers for the subsequent identification of rice food taste and nutritional quality improvement.

## 2. Materials and Methods

### 2.1. Plant Materials and Growth Conditions

The experimental material was Yangnongxiang 28 (no. 20200019, conventional japonica rice). The Yangnongxiang 28 variety was derived from Wuyujing 3/Xudao 3//Huaidao 5. The total growth period of the variety was 149.4 days. Yangnongxiang 28 is a late-maturing mid-japonica rice variety and is suitable for planting in central Jiangsu and Yangzhou.

The rice was grown at the base of Yangzhou University in Yangzhou City, Jiangsu Province. The sowing date of the rice was May 25, 2021, and the blanket seedlings were 25 days old. The rice seedlings were transplanted at the three-leaf and one−heart stage, and four seedlings were planted in each hole. Three treatments were used: high nitrogen and low density (HNLD; high nitrogen: 360 kg·hm^−2^, low density: the row spacing of rice plants was 16 cm × 30 cm), medium nitrogen and medium density (MNMD; medium nitrogen: 270 kg·hm^−2^, medium density: the row spacing of rice plants was 13 cm × 30 cm), and low nitrogen and high density (LNHD; low nitrogen: 270 kg·hm^−2^, high density: the row spacing of rice plants was 10 cm × 30 cm). The field plot area was 12 m^2^ (3 m × 4 m) and the treatments were repeated three times. Ridges were used to isolate the sub-districts in the field, and they were covered with plastic film to ensure separate drainage and irrigation. According to the requirements of the conventional high-yield cultivation of rice, diseases, insect pests, and weeds were kept under control.

### 2.2. Determination of Rice Quality and Taste Value

The rice was harvested in time at the mature stage. Three biological replicates of the rice were taken for each treatment, and they were threshed and dried naturally and stored for 3 months. After the physical and chemical properties were stabilized, the brown rice rate (BR), milled rice rate (MR), head milled rice rate (HMR), and amylose content (AC) were determined according to the national standard of the People’s Republic of China (GB/T17891-2017). The appearance (AP), hardness (HA), viscosity (VI), and balance degree (BD) of the rice were measured using a rice taste meter (STA 1A, Satake, Japan), and the comprehensive scores were calculated.

### 2.3. Metabolite Extraction

The samples of the rice (50 mg) were accurately weighed, and we referred to the method of Xiong et al. (2022) for the extraction of the metabolites and data preprocessing [16]. The metabolites were extracted using a 400 µL methanol/water (4:1, *v*/*v*) solution with 0.02 mg/mL L-2-chlorophenylalanin as an internal standard. The mixture was left to settle at −10 °C. It was then treated using a Wonbio-96c high-throughput tissue crusher (Shanghai wanbo biotechnology Co., Ltd., Shanghai, China) at 50 Hz for 6 min, followed by ultrasound at 40 kHz for 30 min at 5 °C. The samples were placed at −20 °C for 30 min to precipitate the proteins. After centrifugation at 13,000× *g* at 4 °C for 15 min, the supernatants were carefully transferred to sample vials for the LC-MS/MS analysis.

### 2.4. Quality Control Sample

As a part of the system conditioning and quality control process, a pooled quality control sample (QC) was prepared by mixing equal volumes of all the samples. The QC samples were processed and tested in the same manner as the analytic samples. This helped to represent the whole sample set, which was injected at regular intervals in order to monitor the stability of the analysis.

### 2.5. Ultra−High Performance Liquid Chromatography–Mass Spectrometry (UHPLC−MS/MS) Analysis

Chromatographic separation of the metabolites was performed on a Thermo UHPLC system equipped with an ACQUITY UPLC HSS T3 (100 mm × 2.1 mm i.d., 1.8 µm; Waters, Milford, MA, USA). The mobile phases consisted of 0.1% formic acid in water/acetonitrile (95:5, *v*/*v*) (solvent A) and 0.1% formic acid in acetonitrile/isopropanol/water (47.5:47.5:5, *v*/*v*) (solvent B). The solvent gradient changed according to the following conditions: from 0 to 3.5 min, 0% B to 24.5% B (0.4 mL/min); from 3.5 to 5 min, 24.5% B to 65% B (0.4 mL/min); from 5 to 5.5 min, 65% B to 100% B (0.4 mL/min); from 5.5 to 7.4 min, 100% B to 100% B (0.4 mL/min to 0.6 mL/min); from 7.4 to 7.6 min, 100% B to 51.5% B (0.6 mL/min); from 7.6 to 7.8 min, 51.5% B to 0% B (0.6 mL/min to 0.5 mL/min); from 7.8 to 9 min, 0% B to 0% B (0.5 mL/min to 0.4 mL/min); and from 9 to 10 min, 0% B to 0% B (0.4 mL/min) for equilibrating the systems. The sample injection volume was 2 µL and the flow rate was set to 0.4 mL/min. The column temperature was maintained at 40 °C. During the period of analysis, all samples were stored at 4 °C. The mass spectrometric data were collected using a Thermo UHPLC Q Exactive HF-X Mass Spectrometer equipped with an electrospray ionization (ESI) source operating in either the positive or negative ion mode. The optimal conditions were set as follows: heater temperature, 425 °C; capillary temperature, 325 °C; sheath gas flow rate, 50 arb; aux gas flow rate, 13 arb; ion spray voltage floating (ISVF), −3500 V in negative mode and 3500 V in positive mode; and normalized collision energy, 20-40-60 V rolling for MS/MS. The full MS resolution was 60,000, and the MS/MS resolution was 7500. Data acquisition was performed with the data-dependent acquisition mode. Detection was carried out over a mass range of 70–1050 m/z.

### 2.6. Experimental Design and Statistical Analysis

At the maturity stage, rice grain samples were taken from three treatments (every three rice plants were mixed into a rice sample), and each treatment was replicated three times and milled into polished rice with a rice polisher. All of the samples were put into liquid nitrogen and quick-frozen until the rice metabolites were determined.

Multivariate statistical analysis was performed using the ropls (Version 1.6.2, http://bioconductor.org/packages/release/bioc/html/ropls.html (accessed on 15 March 2022) R package from Bioconductor on the Majorbio Cloud Platform (https://cloud.majorbio.com, accessed on 15 March 2022). Principal component analysis (PCA), orthogonal partial least squares discriminate analysis (OPLS-DA), and differential metabolites (DMs) were performed according to the method by Xiong et al. (2022) [16]. Statistically significant differences among groups were selected for VIP values of more than 1 and *p*-values of less than 0.05. Differential metabolites between two groups were summarized, and their biochemical pathways were mapped by metabolic enrichment and pathway analysis based on a database search (KEGG, http://www.genome.jp/kegg/, accessed on 15 March 2022).

The relevant data were collated; calculated as the mean and standard deviation using WPS 2021 software; and analyzed by ANOVA with the output, rice quality, and taste value data using SPSS 18.0 software.

## 3. Results

### 3.1. Analysis of Rice Quality and Taste Value

The amount of nitrogen used and the planting density had certain effects on the rice quality (Table 1). The brown rice rates of LNHD, HNLD, and MNMD had no significant differences. With the increase in nitrogen fertilizer, the milled rice rates and head milled rice rates increased to varying degrees. The milled rice rate of HNLD was 13.85% and 1.89% higher than that of LNHD and MNMD, respectively. The head milled rice rate of HNLD was 32.45% and 6.39% higher than that of LNHD and MNMD, respectively. There were no significant differences in the amylose content, appearance, viscosity, and balance degree among LNHD, HNLD, and MNMD. The hardness of HNLD was the highest and was 7.54% and 6.39% higher than that of LNHD and MNMD, respectively, and the difference was significant. The taste value (TV) of LNHD was the highest and was 4.82% and 1.77% higher than that of HNLD and MNMD, respectively, and the difference was significant.

### 3.2. PCA and PLS−DA

**Figure 1:**
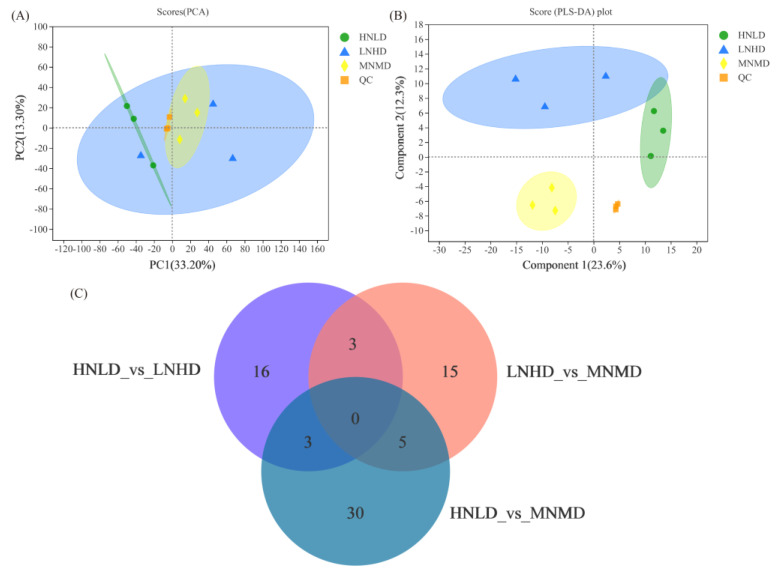
PCA, PLS−DA, and Venn diagram analysis of rice under different nitrogen fertilizers and planting densities: (**A**) PCA, (**B**) PLS−DA, and (**C**) Venn diagram analysis.

To elucidate which metabolites caused the differences in the metabolic profiles under different nitrogen fertilizers and planting densities, non-targeted metabolome analysis was performed on the milled rice treated with LNHD, HNLD, and MNMD. First, PCA was performed on the rice samples. The eigenvalues of the first principal component direction accounted for 33.20% of the total variance value, and the eigenvalues of the second principal component direction accounted for 13.30% of the total variance value (Figure 1A). Then, PLS−DA was performed. The first principal component explained 23.6% of the variance, and the second principal component explained 12.3% of the variance (Figure 1B). This indicates that there were differences in the metabolome of the milled rice tissue under different nitrogen fertilizer and planting density treatments.

### 3.3. Differential Metabolite Analysis

**Figure 2:**
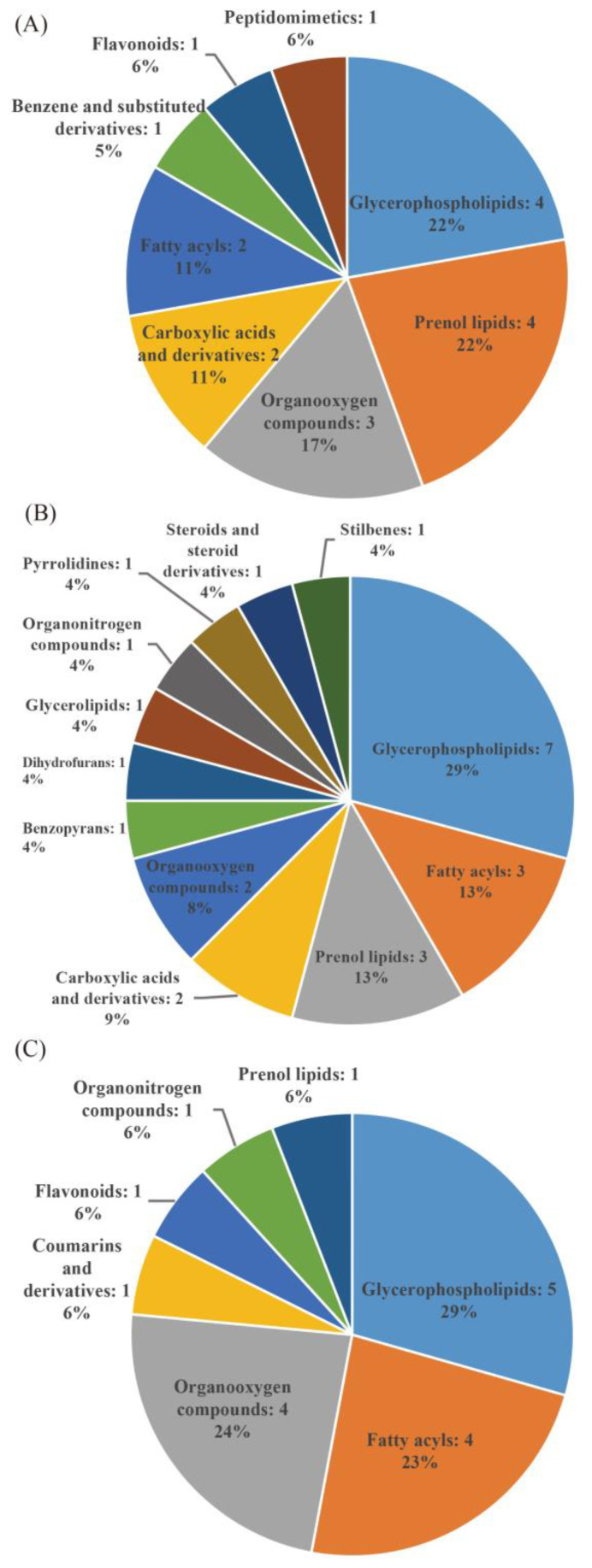
Classification statistics of compounds under different nitrogen fertilizer and density treatments: (**A**) HNLD and LNHD, (**B**) HNLD and MNMD, and (**C**) LNHD and MNMD. According to the number of metabolites, the name of the selected HMDB class (class) and the percentage of metabolites are displayed. The different colors in each pie chart in the figure represent different HMDB classifications, and their areas represent the relative proportion of metabolites in that classification.

The taste quality and nutritional quality of milled rice are important indicators that ultimately determine the rice quality. In this study, 22 significantly different metabolites were found in the milled rice under the HNLD and LNHD conditions (Table S1). The differential metabolites were divided into eight classes, of which glycerophospholipids accounted for 22%, prenol lipids accounted for 22%, organooxygen compounds accounted for 17%, carboxylic acids and derivatives accounted for 11%, fatty acyls accounted for 11%, benzene and substituted derivatives accounted for 5%, flavonoids accounted for 6%, and peptidomimetics accounted for 6% (Figure 2A). A total of 38 significantly different metabolites were found in the milled rice under the HNLD and MNMD conditions (Table S2). The differential metabolites were divided into 12 classes, of which glycerophospholipids accounted for 29%, prenol lipids accounted for 13%, fatty acyls accounted for 13%, carboxylic acids and derivatives accounted for 9%, organooxygen compounds accounted for 8%, benzopyrans accounted for 4%, dihydrofurans accounted for 4%, glycerolipids accounted for 4%, stilbenes accounted for 4%, organonitrogen compounds accounted for 4%, pyrrolidines accounted for 4%, and steroids and steroid derivatives accounted for 4% (Figure 2B). Finally, 23 significantly different metabolites were found in the milled rice under the LNHD and MNMD conditions (Table S3). The differential metabolites were divided into seven classes; glycerophospholipids accounted for 29%, fatty acyls accounted for 23%, organooxygen compounds accounted for 24%, coumarins and derivatives accounted for 6%, flavonoids accounted for 6%, organonitrogen compounds accounted for 6%, and prenol lipids accounted for 6% (Figure 2C). A Venn diagram analysis showed 16 specific metabolites in the HNLD and LNHD comparison group, 15 specific metabolites in the LNHD and MNMD comparison group, and 30 specific metabolites in the HNLD and MNMD comparison group (Figure 1C).

### 3.4. KEGG Analysis

The Kyoto Encyclopedia of Genes and Genomes (KEGG) database is the main public database of metabolic pathways and it can be used in studies of metabolite accumulation in a general network. In this study, we enriched the DMs in each comparison group and classified them into different pathways (Table 2). Beta-alanine metabolism, alpha-linolenic acid metabolism, linoleic acid metabolism, fatty acid biosynthesis, ether lipid metabolism, and glycerophospholipid metabolism were significantly enriched in the HNLD and LNHD groups (*p* < 0.05). Ether lipid metabolism and sphingolipid metabolism were significantly enriched in the HNLD and MNMD groups (*p* < 0.05). The glycosylphosphatidylinositol (GPI)-anchor biosynthesis, phosphatidylinositol signaling system, sphingolipid metabolism, fatty acid biosynthesis, linoleic acid metabolism, and glycerophospholipid metabolism were significantly enriched in the LNHD and MNMD groups (*p* < 0.05). This also showed strong changes in the metabolites in the lipid and amino acid pathways.

### 3.5. Correlation Analysis

**Figure 3:**
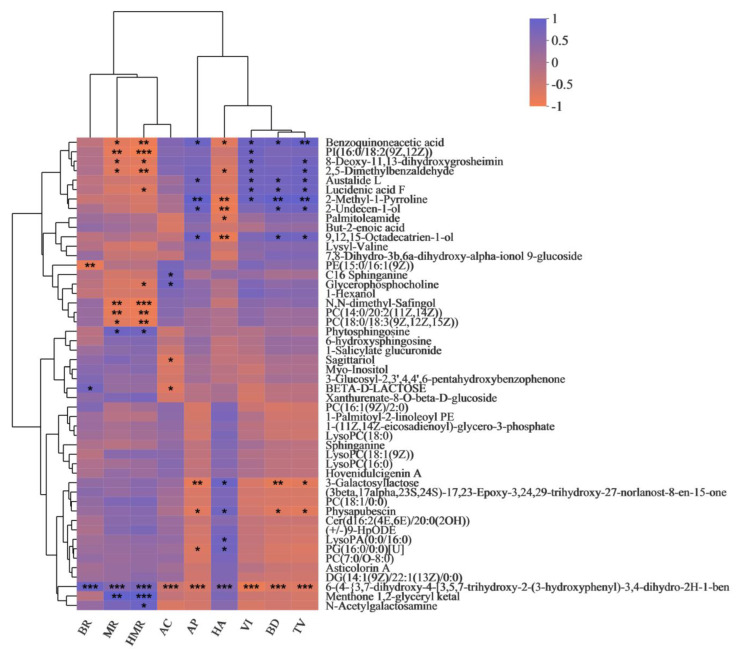
Correlation analysis of BR, MR, HMR, AC, AP, HA, VI, BD, and TV with DMs. The names of the DMs are on the right side, and the names of BR, MR, HMR, AC, AP, HA, VI, BD, and TV are on the bottom. Each grid represents the correlation between the two attributes, and different colors represent the size of the correlation coefficient between the attributes. * *p* < 0.05; ** *p* < 0.01; *** *p* < 0.001.

To better understand the relationship between metabolite composition and rice quality, we performed a correlation analysis of the DMs with BR, MR, HMR, AC, AP, HA, VI, BD, and TV (Figure 3). Benzoquinoneacetic acid and austalide L were significantly positively correlated with AP, VI, BD, and TV. Benzoquinoneacetic acid and 2,5-Dimethylbenzaldehyde were significantly negatively correlated with MR, HMR, and HA. Lucidenic acid F and 2-methyl-1-pyrroline were significantly positively correlated with VI, BD, and TV. C16 sphinganine and glycerophosphocholine were significantly positively correlated with AC. Sagittariol and BETA-D-LACTOSE were significantly negatively correlated with AC. 3-Galactosyllactose and physapubescin were significantly negatively correlated with AP, BD, and TV. Correlation analysis is helpful for gaining a deeper understanding of the metabolic markers associated with rice quality and for further exploring the correlation and formation mechanism of eating quality and nutritional quality.

## 4. Discussion

Rice quality is influenced by genetic factors and cultivation methods [17,18,19]. The brown rice rate, milled rice rate, and head milled rice rate all increased with the increase in nitrogen fertilizer application, which also improved the processing quality of the rice [20]. This study found that the milled rice rate and head milled rate rice under HNLD conditions were higher than those under LNHD and MNMD conditions (Table 1). A higher nitrogen application rate was beneficial to increasing the percentages of brown rice and whole grain rice, but decreased the aspect ratio and amylose content [4]. The processing quality of the rice was improved with high−nitrogen fertilizer application. The effects of planting density on rice quality vary based on type, climatic conditions, and experimental design. An increase in the planting density can significantly improve the processing quality of rice, but it is not conducive to the quality of appearance [21]. However, under the influence of the application of nitrogen, the transplanting density has little effect on rice quality [22]. In one study, the amylose content and taste value of rice were significantly decreased with increases in the nitrogen application level [22,23]. We found that the taste value of the HNLD−treated rice was significantly lower than that in the LNHD treatment, but there was no significant difference in the change in amylose content (Table 1). With increases in the nitrogen application rate, the appearance, viscosity, and balance degree of rice showed a trend of first rising and then falling, and the hardness value showed a trend of first decreasing and then increasing [2]. Previous studies have shown that the condition of a low planting density is beneficial to the grain quality of the rice variety Jingyou 586 [4]. This study found that the appearance, viscosity, and balance degrees of rice under HNLD conditions were lower to varying degrees, but the hardness was increased. Therefore, the HNLD treatment was not conducive to the formation of the rice−eating quality. A reasonable amount of applied nitrogen and an appropriate transplanting density are helpful for improving the rice quality. In the future, more nitrogen fertilizer and density treatments may need to be designed to produce the best planting methods in order to improve rice quality.

Under stress conditions such as drought and cold damage, the contents of glutathione, fatty acids, and pyridoxine in rice are increased [24]. Cultivation methods can affect the accumulation of metabolites in plants, and different growth environments lead to changes in the accumulation and synthesis pathways of metabolites in plants [11,25,26]. This study found that different comparison groups have their own specific metabolites (Figure 1C), and there are differences in the number and proportion of metabolites (Figure 2). As the third most abundant nutrient in rice, lipids are considered to be an important factor affecting the quality of rice [27,28]. Lipids are also important constituents in rice and affect the eating quality [29,30]. In this study, lipid metabolites in different comparison groups were the main components in the grains, and lipids accounted for the highest proportion among all of the metabolites (Figure 2). The correlation results also showed that lipid metabolites were significantly associated with the quality data. For example, sagittariol was significantly negatively correlated with the amylose content; lucidenic acid F was significantly positively correlated with viscosity, balance degree, and taste value; and physapubescin was significantly negatively correlated with appearance, balance degree, and taste value (Figure 3). This is crucial for finding metabolite biomarkers for rice quality formation. In addition, the KEGG metabolic pathway was also mainly enriched in the lipid metabolism pathway (Table 2). Therefore, it is speculated that changes in lipid metabolism may be an important method of improving eating quality characteristics. Flavonoids are important nutrients and have strong antioxidant properties, which can effectively remove oxygen free radicals in plants [16]. Previous studies have shown that there are 11 flavonoid metabolites that are significantly different between Meixiangzhan2 and Qixinzhan [31]. The contents of flavonoids, polyphenols, vitamins, and isoflavones in Meixiangzhan2 are higher than those in Qixinzhan. The most significant differences between the two varieties of milled rice were in the lipids and flavonoids [31]. The flavonoid austalide L was significantly positively correlated with appearance, viscosity, balance degree, and taste value (Figure 3). Whether this is a metabolite for judging good eating quality and nutritional value deserves further in-depth study.

## 5. Conclusions

The amount of nitrogen applied and the planting density of rice significantly affected the rice quality and taste value. Under the influence of nitrogen application, the planting density had little effect on the rice quality. With the increase in nitrogen fertilizer, the milled rice rate and head milled rice rate increased to varying degrees. Among them, the taste value was the highest under low nitrogen conditions. Based on metabolome studies, it was found that lipid metabolites were the main components in grains under the influence of nitrogen application and planting density, and lipid metabolites were strongly correlated with the rice quality data and taste value. The KEGG pathway was also mainly enriched for the lipid metabolism pathways. It is speculated that lipid metabolism may be an important factor affecting the taste quality of rice.

## Figures and Tables

**Table 1:** Differences in taste value, processing quality, and amylose content of rice under different nitrogen fertilizers and densities.

Treatment	Brown Rice Rate (%)	Milled Rice Rate (%)	Head Milled Rice Rate (%)	Amylose Content (%)	Appearance	Hardness	Viscosity	Balance Degree	Taste Value
LNHD	84.46a	63.54b	44.72b	10.71a	8.93a	5.33b	9.03a	8.93a	86.30a
HNLD	84.38a	72.34a	59.23a	10.45a	8.43a	5.73a	8.67a	8.50a	82.33b
MNMD	84.87a	71.00a	55.67a	9.60a	8.80a	5.30b	8.70a	8.77a	84.80ab

Note: Lower-case letters represent the significance of the *p*-value at the 0.05 level.

**Table 2:** KEGG pathways associated with DMs.

Pathway Description	Pathway ID	Ratio_in_Pop	*p*-Value
KEGG pathways between HNLD and LNHD			
Beta-alanine metabolism	map00410	32/4803	0.0264
Alpha-linolenic acid metabolism	map00592	44/4803	0.0362
Linoleic acid metabolism	map00591	28/4803	0.0231
Fatty acid biosynthesis	map00061	58/4803	0.0474
Ether lipid metabolism	map00565	25/4803	0.0207
Glycerophospholipid metabolism	map00564	52/4803	0.0007
KEGG pathways between HNLD and MNMD			
Phenylalanine, tyrosine, and tryptophan biosynthesis	map00400	34/4803	0.0553
Alpha-linolenic acid metabolism	map00592	44/4803	0.071
Ether lipid metabolism	map00565	25/4803	0.0409
Sphingolipid metabolism	map00600	25/4803	0.0409
KEGG pathways between LNHD and MNMD			
Phosphatidylinositol signaling system	map04070	29/4803	0.0357
Inositol phosphate metabolism	map00562	47/4803	0.0573
Sphingolipid metabolism	map00600	25/4803	0.0308
Galactose metabolism	map00052	46/4803	0.0561
Fatty acid biosynthesis	map00061	58/4803	0.0021
Linoleic acid metabolism	map00591	28/4803	0.0345
Alpha-linolenic acid metabolism	map00592	44/4803	0.0537
Glycerophospholipid metabolism	map00564	52/4803	0.0017

Pathway description—KEGG pathway name description; Pathway ID—KEGG pathway ID; Ratio_in_pop—the proportion of metabolites annotated to the background pathway in the background metabolites, the number of the background metabolic set annotated to the left of the diagonal, and the number of KEGG compound IDs of the background metabolic set annotated to all pathways. This function was considered with *p* < 0.05 as a significant enrichment term.

## Data Availability

The data presented in this study are available in this article and supplementary materials.

## Supplementary Materials

The following supporting information can be downloaded at: https://www.mdpi.com/article/10.3390/foods11101508/s1. Table S1: Differential metabolite information between HNLD and LNHD. Table S2: Differential metabolite information between HNLD and MNMD. Table S3: Differential metabolite information between LNHD and MNMD.
